# (Mis)trust among patients seeking unproven stem cell therapies: a qualitative analysis

**DOI:** 10.3389/fmed.2026.1772881

**Published:** 2026-05-13

**Authors:** Omar Kawam, Sara Watson, Xuan Zhu, Shane Shapiro, Jon C. Tilburt, Zubin Master

**Affiliations:** 1 Rutgers Robert Wood Johnson Medical School, Piscataway, NJ, United States; 2 Biomedical Ethics Research Program, Mayo Clinic, Rochester, MN, United States; 3 Robert D. and Patricia E. Kern Center for the Science of Health Care Delivery, Mayo Clinic, Rochester, MN, United States; 4 Department of Orthopedic Surgery, Mayo Clinic, Jacksonville, FL, United States; 5 Division of General Internal Medicine, Mayo Clinic, Scottsdale, AZ, United States; 6 Department of Social Sciences and Health Policy, Division of Public Health Sciences, Wake Forest University School of Medicine, Winston-Salem, NC, United States; 7 Wake Forest Institute for Regenerative Medicine, Wake Forest University School of Medicine, Winston-Salem, NC, United States; 8 Maya Angelou Research Center for Healthy Communities, Wake Forest University School of Medicine, Winston-Salem, NC, United States; 9 Center for Bioethics, Health & Society, Wake Forest University, Winston-Salem, NC, United States

**Keywords:** health behavior, information-seeking behavior, mistrust, qualitative research, stem cells, trust

## Abstract

**Introduction:**

The unproven stem cell intervention (SCI) industry markets stem cell products to patients navigating chronic and serious conditions who seek alternative options when conventional treatments no longer offer relief or improvement. This market is often characterized by providers using deceptive advertising to misinform potential consumers to seek stem cells that have not been shown to be safe and/or effective through clinical studies.

**Objective:**

This study examines how patients describe interpersonal and institutional trust in relation to their interest in and decisions about unproven SCIs among two distinct and divergent chronic disease patient groups: patients with little or no interest in unproven SCIs (low seekers) and those with high interest or have undertaken an unproven SCI (high seekers).

**Methods:**

A semi-structured interview guide was developed deductively and modified inductively based on the Unified Theory of Health Behavior. Qualitative interviews among 36 patients and carers were conduct and transcripts were analyzed using constant comparison based on the principles of grounded theory.

**Results:**

Trust varies across social contexts as low seekers expressed strong reservations about the commercialization elements of the SCI industry placing greater confidence in conventional medicine. In contrast, high seekers questioned the advice of conventional providers and motives of medical regulators. Across both, personal relationships were commonly described as influential in how participants evaluated unproven SCIs.

**Conclusion:**

These findings suggest that communication efforts in clinical settings should account for the relational and emotional dimensions of trust. Interventions aimed at countering misleading information and promoting informed choices should engage trusted messengers and address the broader social and psychological contexts in which patients may seek unproven SCIs.

## Introduction

Many patients with chronic, comorbid diseases have exhausted conventional medical options and are highly motivated to seek out, or undertake, unproven stem cell interventions (SCIs). The unproven SCI industry is a for-profit, direct-to-consumer, international market comprising private clinics that offer FDA unapproved and scientifically unproven stem cell and other cellular and acellular products to older adults with chronic diseases (1, 2). These products are given outside a clinical trial and with little or no safety surveillance. However, reports document patients experiencing physical, financial, and emotional harm (3–6). There are over 2,700 unproven SCI clinics in the U.S., but the industry spans globally (7). Because these services are marketed across many chronic conditions, clinicians in diverse practice settings increasingly face patient questions about unproven SCIs (2, 8–12).

Several factors influence consumer behavior toward unproven SCIs including attitudes toward risk and benefits, science literacy, beliefs, emotions, and trust (13). Patients seeking unproven SCIs are likely to have differing experiences with, and trust among, the diverse individuals, groups, and organizations involved in healthcare practice (e.g., doctors, patient advocacy groups, unproven SCI providers) and clinical research (e.g., biomedical researchers, Institutional Review Boards, pharmaceutical companies, Food and Drug Administration), as well as friends, family, and others (e.g., celebrities, influencers).

Trust is a central feature in healthcare. Trust is directly linked to vulnerability as patients are inherently vulnerable as they place their care and sometimes their lives in the hands of clinicians and organizations with greater power (14). Recent syntheses have clarified distinctions among trust, distrust, mistrust, and trustworthiness definitions in health (15, 16). A patient (the trustor) trusts an actor or institution (trustee) they believe will behave and perform their duties in a competent, considerate, and responsible manner (17). Mistrust is a general attitude of suspicion or unease that a trustee will not meet expectations of the patient or potentially harm them (15). Mistrust tends to refer to general attitudes toward groups of individuals or organizations within healthcare and research, or even the biomedical or healthcare system. Distrust is conceptualized similarly as mistrust, but refers to a specific individual or organization i.e., distrusting a patient’s family doctor (18).

Recent work emphasizes that medical mistrust is multidimensional and can operate across interpersonal and institutional levels, often shaped by fear of exploitation or harm, and by broader systemic inequalities (19); these dynamics can affect how patients communicate with clinicians and how they interpret health information (20). At the level of the clinical encounter, shared decision-making guidance for clinicians underscores that collaborative deliberation depends on a clear invitation, non-abandonment, and the provision of trustworthy information–including transparency about uncertainty–because these features can help protect trust when patients feel vulnerable (21). Empirical studies further suggest that lower trust in the healthcare system is associated with poorer shared decision-making experiences, supporting the idea that system-level trust can condition whether interpersonal communication is feasible and productive (22). This interaction between system trust and clinician communication matters in the unproven SCI context because patients may simultaneously mistrust regulators or pharmaceutical companies while still searching for care in conventional medical systems and private clinics–the latter can appear credible as they present themselves in similar ways to medical systems (e.g., medical credentialing, scientific language, professional clinical presentation) (23). A multi-actor analysis of (mis)trust is therefore needed to understand how patients evaluate these interventions and why clinician–patient conversations about them may succeed or fail.

Most patient perception studies have focused on patients seeking unproven SCIs and the high levels of trust in unproven SCI providers who offer hope and emotional and informational support (13, 24–27). Studies have also shown that patients who undergo unproven SCIs often report frustration with the lack of access to SCIs, a sluggish regulatory process, and feeling dismissed by conventional practitioners, indicating both mistrust of the medical establishment and potentially distrust of specific physicians (13, 24, 28–32). Patients highly motivated to seek out or have undertaken unproven SCIs (defined as “high seekers”) appear to mistrust the biomedical research enterprise considering it overly slow to develop effective treatments for their conditions (13, 29–31, 33), and specifically report believing that the FDA colludes with pharmaceutical companies keeping curative SCIs at bay, permitting the continuous proliferation of treatment drugs with limited efficacy and side-effects (13). While most studies indicate mistrust of the conventional biomedical system, patients have also reported valuing information from their treatment physicians and regulators and prefer obtaining SCIs in their home country due to greater oversight and the fear of exploitation by foreign clinics (13, 34). Several studies have evaluated trust among patients who have sought or undergone unproven SCIs and their social networks including users engaging with and evaluating evidence about unproven SCIs, friends and family who provide informational and emotional support (32, 35, 36), and the potential role of athletes and celebrities (26, 37–40).

These studies highlight the complexity of patient (mis)trust across different actors and what this may mean for the information patients consider and how they evaluate unproven SCIs. However, several gaps remain. First, to the best of our knowledge, all studies focus on patients who have taken, or are strongly considering, an unproven SCI with no study having yet examined patients with little to no interest in unproven SCIs. As a result, there is no direct comparison of trust across these groups. In this study, we stratify chronic disease patients into low seekers (patients with little to no interest in unproven SCIs) and high seekers (patients with high interest or prior use of unproven SCIs) to compare trust profiles across the two cohorts. Second, most studies infer trust indirectly (e.g., from the acceptance of information, slowness of regulatory system) rather than directly eliciting trust in specific actors and institutions. The present analysis directly examines trust across multiple actors and institutions. Third, most studies capturing patient attitudes are when travel from high-income countries was required when seeking unproven SCIs and very few studies examined the attitudes of U.S. patients. Given the unproven SCI market has shifted from predominantly cross-border care toward a substantial domestic U.S. marketplace, contemporary U.S.-based evidence is needed to understand trust dynamics in the setting where many patients now encounter and purchase these interventions.

Our study aims to remedy these gaps by directly assessing (mis)trust in multiple professional and personal actors involved in unproven SCIs among two diverse populations – low seekers (patients with little to no interest in unproven SCIs) and high seekers (patients highly interested in or who previously had undertaken unproven SCIs) through qualitative interviews of U.S. chronic disease patients. The results published here are part of a larger study to understand the factors and their impact toward undertaking an unproven SCI (13). The theoretical foundation, interview guide, study population and recruitment strategy, data collection, and qualitative analysis remain consistent with our previous report. Our prior report identified three themes that impacted patients’ willingness to undertake unproven SCIs: information sources/knowledge; attitudes about safety, efficacy, and expectations; and desperation/affect (13). The current manuscript represents results of a single and complex theme of (mis)trust separated into seven subthemes: SCI Clinic Quality, Financial Motives & SCI Clinic Legitimacy, (Mis)Trust of SCI Providers, (Mis)Trust of the FDA, (Mis)Trust of Pharmaceutical Companies, (Dis)Trust of Physicians, and Trust in Friends, Family and Celebrities.

## Materials and methods

We conducted qualitative interviews with patients seeking unproven SCIs and their carers, defined as either an immediate family member(s) or non-family caregiver(s) fulfilling a primary caregiving role. The present study covers a single theme of (mis)trust among multiple actors and institutions in the unproven SCI context. The data presented here is distinct and has not been published with prior research reporting the themes of knowledge, attitudes on safety, efficacy and expectations, and desperation (13). The theme of (mis)trust was separated into its own publication given its complexity and to enhance readability.

The following sections provide a brief summary of study methods. Methodological details and justifications have previously been described in detail elsewhere (13). The Supplementary material provides additional methodological details on patient recruitment, interview guide development, and conducting interviews; a researchers reflexivity statement; and semi-structured interview guide. All procedures were performed in compliance and approved 01/21/2021 by the Mayo Clinic IRB (#20-013345).

### Recruitment

U.S.-based patients and carers registered in the Mayo Clinic Regenerative Medicine Consult Service database were purposively sampled to maximize variation in primary health concern, geography, race and ethnicity, rurality, gender, and across a wide range of chronic conditions. Research coordinators telephoned or emailed eligible participants and outlined study aims, reviewed privacy safeguards and withdrawal rights, and determined SCI seeking status as high or low based on responses to three survey items. Those who completed interviews were remunerated $25 for their participation.

### Interview guide development and conducting interviews

An *a priori* semi-structured guide informed by Unified Theory of Health Behavior (41) was developed deductively incorporating additional variables relevant to unproven stem cell-seeking behavior. Questions related to trust in medical institutions were adapted from existing studies and contextualized to assess trust in regulatory bodies (e.g., the U.S. Food and Drug Administration), biomedical researchers, clinicians and their affiliated institutions, as well as providers offering unproven SCIs (42, 43). The interview guide was modified inductively through four pilot interviews. Verbal consent was obtained from all participants. All interviews were audio recorded and professionally transcribed. Interviews continued until thematic saturation was achieved. A reflexivity statement is provided in the Supplementary material.

### Qualitative analysis and reporting

Three investigators documented analytic reflections on five transcripts and collaboratively conducted open coding to develop a preliminary codebook. This codebook was subsequently refined through analysis of five additional transcripts with discrepancies resolved through consensus. The remaining interviews were single-coded and subjected to secondary review with unresolved excerpts discussed in regular team meetings. Data analysis was guided by a constant comparative grounded theory approach. Intra and inter-family contrasts to surface power dynamics and contradictions were considered. Reporting adheres to the COREQ checklist (44).

## Results

### Demographics

In total, 36 individuals participated in this research study, consisting of 28 patients and 8 carers (Table 1). The study population was predominantly male and included a higher proportion of participants who identified as White and Black/African American than U.S. national demographics. Participants were categorized into two groups, High Seekers and Low Seekers, based on their engagement and interest in unproven SCIs. The seeking status of carers was aligned with the corresponding patient’s status. Participants reported residence across 17 US states, with most living in urban settings. The study population presented a diverse range of medical conditions.

**TABLE 1:** Participant demographics.

Sociodemographics	Low seekers		High seekers		Total participants	
	*N*	Percent	*N*	Percent	*N*	Percent
Patients	15	54%	13	46%	28	100%
Carers	4	50%	4	50%	8	100%
Patient age						
Mean	59.3	–	63.8	–	61.4	–
Range	33–78	–	37–94	–	33–94	–
Sex						
Male	12	63%	12	71%	24	67%
Female	7	37%	5	29%	12	33%
aHighest level of education attained						
HS diploma/GED	1	5%	5	29%	6	17%
Some college	7	37%	4	24%	11	31%
Associate degree	1	5%	0	0%	1	3%
Bachelor’s degree	3	16%	4	24%	7	19%
Master’s degree	2	11%	2	12%	4	11%
Professional or doctorate degree	3	16%	2	12%	5	14%
Race						
White	12	63%	13	76%	25	69%
Black/African American	6	32%	4	24%	10	28%
Two or more races	1	5%	0	0%	1	3%
bEthnicity						
Hispanic or Latino	3	16%	1	6%	4	11%
Not Hispanic or Latino	15	79%	15	88%	30	83%
cRural-urban commuting area (RUCA) classification						
Urban	17	89%	15	88%	32	89%
Large rural	2	11%	2	12%	4	11%
States	AZ(2); CA(2); CO(2); HI(1); IL(1); MA(1); MI(2); MN(2); MO(1); NC(1); PA(2); SC(1); TX(1)			AZ(1); CA(5); FL(3); IA(1); IL(1); MD(1); MI(1); MN(2); WI(2)		
Conditions	Chronic Kidney Disease(2); ^e^COPD(2); Heart Failure(2); Hemorrhagic Stroke(3); Kidney Failure(1); Neuropathy/Peripheral Neuropathy(2); Spinal Cord Injury(3)			^d^ALS(1); Spinal Disk Issues(1); Glaucoma/Blindness(1); Ischemic Stroke(2); Macular Degeneration(1); Microfiber Neuropathy(1); Neuromuscular Issues(1); Neuropathy(1); Knee Osteoarthritis(1); Renal/Heart Failure(1); Rheumatoid Arthritis(1); Spinal Cord Injury(1)		

^a^Missing data: highest level of education: 2 low seekers did not provide.

^b^Missing data: ethnicity for one low and one high seeker was not obtained.

^c^A four category classification system was used based on RUCA scores.

^d^Amyotrophic lateral sclerosis.

^e^Chronic obstructive pulmonary disease.

#### Trust

**FIGURE 1:**
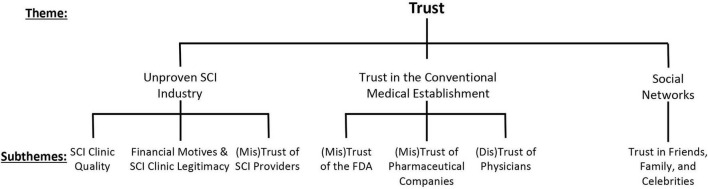
Theme of trust with 7 subthemes based on the unproven SCI industry, conventional medical establishment, and social networks.

We report observations on the theme of (mis)trust into 3 social spaces and 7 subthemes: (1) SCI clinic quality, (2) financial motives and SCI clinic legitimacy, (3) SCI providers, (4) FDA, (5) pharmaceutical companies, (6) physicians, and (7) friends, family and celebrities (Figure 1). Participants described trust in various actors and institutions in relation to the information sources they relied on and their views of unproven SCIs.

#### Trust in the unproven SCI industry

Participants’ views toward clinic quality, their financial motivations, and the providers of unproven SCIs shaped their attitudes toward trusting or mistrusting the industry, which participants described as relevant to how they evaluated unproven SCIs and whether they remained open to pursuing them.

##### Subtheme 1: SCI clinic quality

The quality of the clinics described by participants included features about the operation and appearance of the physical clinic including communication, cleanliness, and friendliness of clinician and staff. Many high seekers reported perceiving clinic quality as marker of its legitimacy. The timeliness of care and the professionalism of staff, nurses, and recruiters contributed to participant perception of clinics as being well-managed and trustworthy. These markers included whether the clinic was proactive in answering phone calls; responding to questions about costs and expected outcomes; and being transparent about the SCI procedure. Specifically, one participant expressed how impressed they were with one clinic stating, “The quality of the clinic was reasonably high end…This was implying that the [clinician] was making reasonable income, so it wasn’t [a] mom-and-pop operation” (Participant 5, Patient, High Seeker, Unspecified Neuromuscular Issue).

Several clinics have been known to offer adjuvant therapies alongside unproven SCIs. Among those that did, some high seekers who underwent unproven SCIs expressed that the additional luxury treatments (e.g., pedicures, access to resort facilities, vitamin infusions) were complementary to the experience, though it was unclear if this increased their perception of the effectiveness of the SCI specifically. Some high seekers were not looking for a therapy alone, but a holistic experience that could aid them in emotional well-being above physical healing. One carer described their experiences at a for-profit clinic for the patient who was suffering with depression and received a total care package: “The first thing the [nurse] does is turn on the computer and put on relaxing, soothing music and starts to tell my mom how beautiful life is, how she should thank her legs…Then, they gave her a lotta vitamins over the course of the day to prepare the body. They took tests to make sure she was able to handle the hyperbaric chamber. They told her to hold the cells themselves, in her hands, to warm them up. It was very holistic. Whether or not that works or–I guess it’s all matter of opinion and experience. Even if it didn’t work, it made my mom believe there was a chance. She was happy. She believed she was in good hands and that this was going to work” (Participant 6, Carer of High Seeker with ALS).

Contrarily, many participants with low interest in seeking unproven SCIs expressed a baseline mistrust of unproven SCI providers and were less likely to visit clinics for a consultation and thus could not comment on clinic quality. For those low seekers who did visit a clinic, markers of a poorly run establishment reinforced their mistrust. Additionally, participants with low interests in unproven SCIs were often unimpressed with “strip-mall” clinics that offer spa treatments as part of the therapeutic experience. One participant described the clinic in an upscale neighborhood as follows: “There’s stem cell people who are doing this red blood thing in his knee. It’s in a clinic like–I hate to say it–like a strip mall kinda thing. Like a spa, like a day spa to get your stem cells…You have your liposuction, you have your day spas, you have your stem cells. It’s very available here. It’s right down the road, and everybody does it. It’s all part of the plastic, puffed up, Botox. It’s like Botox is every corner, so why not stem cells on every corner” (Participant 29, Carer of Low Seeker with Heart Failure).

Such low seekers reported that they sought their care for specific medical conditions and did not see the relevance of adjunct therapies and SCIs.

##### Subtheme 2: Financial motives and SCI clinic legitimacy

Most high seekers reported that, in principle, they do not have a problem paying for experimental treatments. For some, costlier SCIs were reported as being higher quality and more trustworthy than other clinics. One participant explained, “You know you got to pay for it. If it says it does what it does, then you do what you have to do. You pay for it. It’s not gonna be cheap” (Participant 19, Patient, High Seeker, Ischemic Stroke).

Contrarily, many low seekers reported perceiving the high costs of unproven SCIs as a reason to mistrust the legitimacy of SCI clinics. Most low seekers expressed financial motivations of clinics as a major cause of mistrust. Such perceptions were derived from experiences where participants reported seeing sales advertisements of clinic websites or through interactions with providers where participants reported feeling they were being “sold” unproven SCIs. Low seekers explained that they did not perceive their healthcare transactionally and reported uneasiness when clinics foregrounded financial motivations. This rationale clearly manifested when some participants reported clinics were bargaining prices or offering promotional deals, such as buy-one SCI infusion to get another half price. One participant explained that a SCI provider was negotiating prices: “I’ve never been at a doctor’s office where once he’s told me that the cost of [the treatment] was $28,000 to $31,000, ‘Okay, well, let me see if I can help you out a little there.’ [The provider and recruiter] leave the room and come back, and now, it goes from $28,000 down to $21,000. It almost came across to me as something I’m used to doing when I lived in [state] selling Lamborghinis and Maseratis where I’m negotiating with people about money when they can’t afford to pay what they wanna buy. ‘I’m like…first of all, sir, how can you just drop the price of something like that that quick?’ That was a major skepticism right there. Once that took place–it went from 31 to 28 down to 21 to where, ‘Okay, well, how about we just treat your hands and not treat your feet?’ I’m like, ‘Well, what kind of doctor would only want to treat part of your body based on how much it costs and the affordability of what the person can pay?’ (Participant 14, Patient, Low Seeker, Peripheral Neuropathy).”

The participant further reported that the doctor tried to guilt him into undertaking the unproven SCI: “‘Well, if you can’t afford it, then this is your last option. You’ve tried everything else and nothing else is working, so obviously you are here because we are your last resort. Why don’t we go, just go ahead and just agree to get this done? We can try to get you back to feeling better again” (Participant 14, Patient, Low Seeker, Peripheral Neuropathy). Many low seekers mentioned that discussions around finances significantly reduced trust among unproven SCI providers and reduced credibility of the procedures.

##### Subtheme 3: (Mis)trust of SCI providers

Many high seekers reported wanting and receiving personal attention from providers. This may come in the form of the provider asking about their physical, but also emotional well-being, making themselves available to patients, demonstrating a sincere desire to assist them where other physicians could or have not, and showing respect by permitting participants to exercise autonomy and explaining that the final decision about pursuing an unproven SCI was up to them. One participant explains “He treated me more like a person, and not just somebody coming in to pay him some money…So I think he cared” (Participant 2, Patient, High Seeker, Rheumatoid Arthritis). A few participants reported having ongoing relationships with their providers as one participant explained “I still interact with them [the clinic] on a WhatsApp basis and send pictures and updates of how I’m doing. They have, since, been very caring and reaching out to check on me.” (Participant 4, Patient, High Seeker, ALS).

When we asked patients about their interest in the qualifications of unproven SCI providers, most high seekers reported that they were interested in the information but did not seem to know the provider’s area of specialty when prompted. We observed that most high seekers were unfazed by a generalist or non-stem cell expert offering SCIs. They expressed satisfaction when observing some medical degree (usually an MD) and provider explanations of their experience with SCIs. High seekers did not report being interested in providers’ affiliations with known institutions or research contributions. A few low seekers who explored unproven options also exhibited uncertainty regarding providers’ qualifications. When asked about the qualifications of a provider, one low seeker responded: “Well, I didn’t look. I asked them, and I talked to them about it on the phone…I think I was satisfied that the doctors were who they said they were as far as being knowledgeable and all that” (Participant 27, Patient, Low Seeker, COPD).

Accurately identifying proper provider qualifications was uncommon among most high seekers and some low seekers. However, most low seekers reported recognizing that only specialists should perform SCIs. They expressed that they did not consider SCIs as a treatment that can be performed by a generalist clinician, but a technical procedure that should be done by an expert specialized in stem cell therapies. One low seeker expressed skepticism toward a one-shot cure-all stating, “It’s not going to be as simple as giving a person a shot with stem cells. I mean I think there will be different procedures for different illnesses” (Participant 1, Patient, Low Seeker, Chronic Kidney Disease), suggesting that different administration procedures necessitate clinical specialization. Some low seekers also reported that they did not consider specialists who operated outside their area of specialty as serious about SCIs and mistrusted them.

#### Trust in the conventional medical establishment

Participants’ attitudes toward governmental regulatory bodies like the FDA, pharmaceutical companies, biomedical research institutions, and their relationships with medical providers influenced how patients appraised the unproven SCI industry. High and low seekers alike reported having reservations toward governmental regulation, but high seekers were more likely to have mistrust or distrust with actors in conventional medicine.

##### Subtheme 4: (Mis)trust of FDA

Most low and high seekers expressed some awareness of the FDA and its involvement in regulating drugs and devices, but their trust and interest in its regulatory recommendations were modest to low. For many high seekers and some low seekers, the FDA was not a major factor in their decision-making about SCIs. Most participants reported that they believed the FDA is a bureaucratic body that slows down research and approval of SCIs. Although most participants mistrusted the FDA, a negative, disaffected attitude was more commonly expressed amongst high seekers. For example, one participant stated, “FDA is holding up a lot of research” and mentioned hearing this rhetoric from their for-profit SCI providers: “[The providers] said that the FDA took so long. They bypassed a lot of things regarding the FDA, and they just went on and started working on their own” (Participant 22, Patient, High Seeker, Glaucoma and Blindness). Many participants similarly voiced a desire to speed up research or expedite certain regulatory hurdles to access unproven SCIs, especially in the context of clinical trials.

Most high seekers reported that they do not mind skipping these regulatory processes and go to for-profit clinics instead. These participants often reported believing the SCIs were natural and safe or effective enough and worth trying and disregarded the FDA’s opinion: “I know that they [*referring to SCIs*] are not FDA approved or all that, but I don’t know. They’re natural. They’re stem cells. I wasn’t afraid that anything negative was going to happen to my body” (Participant 2, Patient, High Seeker, Rheumatoid Arthritis).

Many high seekers expressed conspiratorial views that the FDA was colluding with Big Pharma to obstruct research and the dissemination of SCIs. One patient discussed their reservations stating, “From my perspective, FDA is not necessarily [a] super ethical organization. FDA is protecting from one point of view, big business interests, and patients are secondary” (Participant 5, Patient, High Seeker, Unspecified Neuromuscular Issue)

A minority of high seekers, however, recognized the FDA as a useful body, but their desire to get unproven SCIs made the FDA’s opinion less worthy of consideration. Other high seekers who reported caring about regulatory approval, accreditation, and the source of stem cells mentioned that they look for regulatory approval from other bodies (e.g., ISO 9000 accreditation) and not just the FDA, which they expressed as being too restrictive or faulty.

While many low seekers expressed not being fully willing to endorse the FDA, many also stated that the FDA plays a useful and important role in determining safety and efficacy of drugs and medical interventions. Some patients relied on their physicians to confirm the safety of a procedure from the FDA stating, “Well, I can look it up on my own to see if something is FDA approved, but I have high trust in my physicians to tell me that. If they say it’s FDA approved, I have no reason to doubt that” (Participant 16, Patient, Low Seeker, Chronic Kidney Disease).

We observed that participants who expressed trust in the FDA, importance in ethical review of experimental SCIs, and were aware of participant rights and regulatory norms about healthcare were less likely to trust for-profit clinics and were willing to undergo a SCI only after FDA approval. One low seeker equivocated on the role the FDA played in their decision stating, “I don’t have high trust in [the FDA]. They have a lot of rules and regulations…but I agree with the FDA on certain things, and I disagree with the FDA on certain things. That’s all. They are certainly helpful in determining my decision” (Participant 35, Patient, Low Seeker, COPD)

##### Subtheme 5: (Mis)trust of pharmaceutical companies

We observed that most high seekers often developed generalized views about the medical establishment based on a mix of their self-reported experiences with physicians, media reports, and political beliefs that foster mistrust in these organizations. As mentioned earlier, these views included conspiratorial beliefs that government and Big Pharma were colluding to obstruct progress in SCIs, based on the assumption that widespread access to SCIs would undermine Big Pharma’s profits. These participants reported that SCIs are curative, and thus pharmaceutical companies cannot make money off them and continue to sell band-aid treatments as one participant explained: “I just think that they are probably maybe not FDA approved because it could help a lot of ailments, and the pharmaceutical companies might not make as much money as they’d like. *[Laughter]* To me, otherwise, I can’t think of any logical reason why they [SCIs] wouldn’t be, why insurance companies wouldn’t cover something like this when there’s a good chance of them working with minimal side effects” (Participant 2, Patient, High Seeker, Rheumatoid Arthritis).

Notably, one high seeker reported trusting the scientists and physicians doing cutting-edge stem cell research, but with the caveat that they were intertwined with government interference that would stifle their work: “I think that researchers and scientists should be the leaders in medical care, not FDA. FDA has its place too, but not in the place of the leading in healthcare. That is out of their lane…I don’t think that that’s the government’s policy. I mean, it’s not the government that should be [recommending what is safe and effective]…I think that the government should work closely with the scientists and the researchers, and the researchers should dictate whether or not it’s 90% effective, whatever the situation is [what] the research says. The government should share that with the people” (Participant 22, Patient, High Seeker, Glaucoma and Blindness).

Low seekers, however, expressed reduced trust of pharmaceutical companies because of their financial motives, but did not share the same degree of conspiratorial thinking as high seekers about pharmaceutical companies and the FDA.

##### Subtheme 6: (Dis)trust of physicians

Participants frequently described discussing unproven SCIs with their physicians. Many high seekers reported that they explored multiple therapeutic avenues including seeking clinical trials to little or no avail. Some mentioned being exhausted from their conventional clinical care experience, including with their physicians, and were seeking alternatives. Other high seekers expressed experiencing emotional harm from physicians. Some participants reported physicians being dismissive of their concerns, derogatory, or displaying no empathy. One participant described their experience stating: “*[Sharing news of my condition]* was not difficult for [*sic*] them at all. That’s what is so shocking. They did it with no empathy, no compassion, no caring about how I was going to receive it. Just dropped it on me like burning coals” (Participant 4, Patient, High Seeker, ALS).

High seekers frequently reported that their physicians were either incompetent, or in the context of stem cells, were too narrow and “by the book,” not being interested or sufficiently educated about stem cell therapies to make adequately informed recommendations.

Another participant shared their attitudes about their doctors: “I found that physicians have very narrow minds. For example, I got 30 slides from my MRI/PET scan a few weeks ago. I went to [a] neurologist, and she even didn’t want to look at the slides. She just wanted to look at [the] regular MRI. She cannot think outside of the box that she was trained in. My perception is that doctors have been trained for a long time, and it is extremely difficult for them to step outside of the box that they were operating in the past” (Participant 5, Patient, High Seeker, Unspecified Neuromuscular Issue).

Assuming their physicians were not valuable resources for care and unwilling to search for treatments, high seekers expressed looking to other sources for care. This included other legitimate hospitals and research institutions, but if they did not find support, they resorted to unproven SCI clinics.

Low seekers, however, expressed being more trusting of their primary care providers and specialists. Even when they did not receive additional treatment options, these participants reported trusting physician recommendations and would not feel inclined to seek care from clinics with less reputable treatment offerings. One participant described taking their physician’s advice not to be duped by the for-profit provider explaining: “[My doctor] said, ‘I tell you what you do.’ She said, ‘You go get your $15,000 [for the SCI] and bring it here and give it to me.’ She said, ‘I’m going to take it out and spend it.’ She said, ‘You’re going to get just as much good out of me spending it as you will over there.’ She said, ‘We are as good as anybody around at knowing what is going on.’ She said, ‘Be real careful.’ She said, ‘I’m not going to tell you not to spend your money,’ she said, ‘but be real careful you’re going to spend your money and not get anything out of it.”’ (Participant 33, Patient, Low Seeker, Spinal Cord Injury).

Two other low seekers expressed their doubt in unproven SCIs because their physicians did not recommend it: “Why aren’t…physicians recommending this? That was the key. Because the physicians aren’t, and the physicians are holding back. If it’s not coming from the physicians and it’s just coming from the media…We came to all the physicians. They didn’t bring this up to us…. All of them independently poo-poo’d the idea.” (Participant 29, Carer of Low Seeker with Heart Failure).

#### Personal connections and social ties

##### Subtheme 7: Trust in friends, family, and celebrities

Most participants reported having family and friends who support them on their healthcare journey. This support ranged from tending to daily medical needs to having occasional discussions about health. Participants reported that they held recommendations of family and friends in high regard if they trusted these individuals. Most high seekers who received recommendations for unproven SCIs from a family or friend who had positive reviews were more likely to consider the intervention. The opposite was observed for low seekers if the family or friend offered a negative opinion of SCIs. One patient discussed their trust in their son as he supported the patient in their SCI journey: “Because [my son] is good at researching, and he is thorough, I trust him with all my heart. He was the most motivated about the stem cells from the research he had done. I didn’t have anyone else advocating for me. I wasn’t, mentally, able to do it, so he did…The doctors, to me, were being very pessimistic. My son was optimistic and found something active that I could do that gave me hope. I wasn’t ready to give up hope. I was starting to slide into no hope, but I owe it to my family to do whatever I can. That’s why I was willing to do whatever he felt I should do” (Participant 4, Patient, High Seeker, ALS).

Additionally, high seekers also reported that in many cases, the patient’s family and friends supported the choices made by the patient and did not offer recommendations contrary to the patient’s desire to pursue an unproven SCI. One patient mentioned, “I put it on Facebook. My friends all agreed. There was not one negative comment about it” (Participant 4, Patient, High Seeker, ALS) In many cases, friends and family supported participant decisions, regardless of whether that is getting a SCI or not. Another participant explained: “My friend whose cousin had this done is a chiropractor, and I just talked to him about it a little bit, and he basically just said, ‘You have nothing to lose.’ I talked to him for some advice, and, well, my husband, of course, and he pretty much had the same idea as [the chiropractor]. You have nothing to lose about it, and that was pretty much it” (Participant 2, Patient, High Seeker, Rheumatoid Arthritis).

Some high seekers also reported having colleagues, co-workers, or bosses who they held in high regard due to business success, esteem for their intellect, or innovative thinking. One patient explained their decision to on having undertaken an unproven SCI stating: “I trusted my VP of marketing that he knows what he is doing. He was responsible for growing < Company’s > business [from] $1 million to $5 billion or something like this. The other guy is teaching [how to use a technological device] all over the world. I trusted him. I trusted the recommendation for Dr. < Name >. This was the biggest thing for reducing the anxiety for the outcome” (Participant 5, Patient, High Seeker, Unspecified Neuromuscular Issue).

Few high seekers also reported trusting the advice of acquaintances and online celebrities, i.e., Joe Rogan or Mel Gibson, who promoted unproven SCIs. We also observed that some high seekers were trusting of people they reported being impressed by and followed their recommendations. These participants expressed they were less likely to believe in the conflicts of interest or lapses in judgment of people they highly trust. Contrarily, low seekers did not mention celebrities in their social network who were influential.

While low seekers also reported using their social networks for support in decision making, they were inclined to accept advice that discouraged unproven SCI use. When they received disapproval or heard bad reviews from trusted friends or family, low seekers expressed being less likely to pursue unproven SCIs. One participant explained the influence of his children on avoiding an unproven SCI stating: “Even my sons were very–well, one’s a medical engineer. He designs and makes implants for knees and for hearts and for all of that. He’s got a minor in biology, and he’s very smart. My younger son also. They were against it” (Participant 35, Patient, Low Seeker, COPD).

However, we observed that ambivalent attitudes toward unproven SCIs from friends and family kept participants uncertain about what to do if they did not already have a decision made.

## Discussion

Our thematic analysis demonstrates that trust operates along multiple dimensions with distinct profiles between low and high SCI seekers. These findings suggest that trust may be an important contextual factor in how patients evaluate unproven SCIs and may have implications for patient communication.

### (Mis)trust in the unproven SCI industry and its markers of legitimacy

Our data suggests key differences among high and low seekers surrounding (mis)trusting the unproven SCI industry, including SCI providers. High seekers consider clinic quality (e.g., punctuality, friendliness of staff, and cleanliness) and price as markers legitimizing the quality of products and services offered by unproven SCI clinics, consistent with previous studies (25–27, 35). We previously found that both low and high seekers often engage in peripheral processing when moving toward a preconceived behavioral intention (13). Thus, heuristic markers of legitimacy may be especially influential for high seekers interested in pursuing unproven SCIs when navigating a path where traditional markers of legitimacy, trust, and medical authority are absent (45). It is well-documented that providers and clinics use tokens to scientifically legitimize the practice of unproven SCIs, including the use of institutional review boards (IRBs), cross-branding with legitimate medical institutions, scientific publications, polished seminars, utilization of ClinicalTrials.gov, and stem cell training courses (23, 46–49). Because these markers resemble safeguards and credibility cues associated with evidence-based care, some patients may interpret them as proof that an intervention is safe, effective, or approved by regulators even when these signals are incomplete, easily misunderstood, or used in ways that do not require rigorous evidence. Such legitimacy cues may also complicate patient-clinician conversations and shared decision-making because clinicians may need to explain why these credibility markers do not substitute for strong clinical evidence and clear regulatory authorization, especially when clinical outcomes remain uncertain.

Our results also demonstrate that low seekers mistrust the unproven SCI industry and its players and have greater trust in conventional medical physicians and institutions. This mistrust, including paying for unproven therapies, could be premised on skepticism toward commercialized health care and medical profiteering and the belief that health care should be reasonably priced (50). Additionally, low seekers may have higher levels of trust in actors within the medical health care establishment. Similar results are seen among other studies that show that individuals with high trust in conventional medicine are less interested and have fewer CAM consultations whereas mistrust in conventional medicine, similar to high seekers of unproven SCIs, is correlated with increased CAM consultations (51). Similarly, vaccine hesitancy has also been shown to be strongly associated with mistrusting conventional medicine (52).

Given low seekers’ general mistrust of the unproven SCI industry, it is not surprising that many did not follow up with clinics or speak with providers. In contrast, high seekers showed a considerable level of trust in unproven SCI providers, valued personal attention, emotional support, and ongoing relationship with providers. This pattern aligns with previous anthropological work emphasizing the relational and emotional dimensions of medical encounters among patient-SCI provider encounters (25, 31). Given this trust, it is not surprising that high seekers sought out and believed information offered by SCI providers (13). Patients desire clinicians to be respectful, listen, be interested in their feelings, caring, and non-judgmental (53), especially in terms of communicating about unproven SCIs (10, 11). Similarly, patients interested in CAM therapies praise CAM practitioners for personal attention and having genuine interest in their care compared to conventional medicine clinicians (54, 55).

These results suggest that low seekers place greater emphasis and institutional trust in conventional medicine whereas high seekers may have greater mistrust in conventional medicine and high trust in SCI providers. High seekers of SCIs desire personal attention and may customize an epistemic framework to align with their preferences. These observations suggest that institutional trust may be an important contextual factor in whether patients seek unproven SCIs.

### (Mis)trust in conventional medicine and research

Our results suggest fewer remarkable differences among high and low seekers surrounding (mis)trusting of actors and their institutions within conventional medicine and research compared to the unproven SCI industry. As explained above, low seekers highly trust conventional medical practitioners whereas high seekers expressed challenges conversing with conventional providers driving them toward unproven SCI providers, which is corroborated by multiple previous studies (28, 30, 32). However, both groups of patients expressed misgivings surrounding the FDA and pharmaceutical companies.

Patients reported appreciating FDA advice, but low seekers accepted FDA’s regulatory role as safety gatekeeper whereas high seekers ignored FDA recommendations and questioned the need for regulations. Generally, Americans have low levels of trust in the FDA with greater mistrust associated with being female, living in a rural community, conservative political leaning, self-reported worse health, reduced satisfaction with health care, and less attention to health and science news (56, 57). Specifically, patients who seek unproven SCIs seem to mistrust regulatory agencies considering them sluggish and overly bureaucratic (24, 29, 31), but these attitudes come with reservations as Australian patients prefer domestic SCIs fearing exploitation from foreign clinics (34) and US patients who highly seek unproven SCIs still appreciate information from the FDA (13).

In our study, both high and low seekers mistrust pharmaceutical companies, but some high seekers exhibited conspiratorial thinking about a collusion between FDA and pharmaceutical companies (13). Generally, Americans have low levels of trust in pharmaceutical companies likely due to profit motivations, off-label marketing, overcharging government, and concealing data (58). These results suggest that FDA regulatory warnings and “not approved” disclosures may be informative for low seekers, but may be discounted by high seekers. The results also suggest that information about regulatory oversight may be more effective when delivered through trusted intermediaries i.e., clinicians and professional societies (59). These trusted sources can translate the meaning of “not approved” in terms of uncertainty about safety and effectiveness. Clinicians may also need to clarify why common legitimacy cues cited by clinics (e.g., IRB review, trial registries, and scientific publications) do not necessarily indicate rigorous evidence of clinical benefit.

### Trust in social networks

Our findings suggest that high and low seekers had high levels of trust in friends and family who supported their decision to obtain or not obtain an unproven SCI, respectively. Previous research has shown that patients considering unproven SCIs are strongly influenced by friends, family, and other patients who are major sources of information and recommendation (12, 13, 25, 28, 31). This finding is corroborated by prior research demonstrating the centrality of personal networks at influencing patient decisions, including among patients seeking CAM therapies (60–62).

Interestingly, neither high nor low seekers in this study mentioned friends or family providing information incongruent with their decision to pursue or refrain from undertaking an unproven SCI, respectively. One explanation for this observation is seen as a form of protective buffering. Protective buffering is a relationship-focused coping strategy where family and friends deliberately suppress or downplay worries, doubts, or negative information so that the patient can maintain optimism, hope, and a sense of control surrounding their illness (63). Protective buffering is seen among patients and their relatives and friends with chronic diseases and cancer (64, 65). Some close friends and family of high seekers may buffer their concerns if the patient has a high desire toward an unproven SCI for fear that they may upset the patient, add to their struggle, or appear unsupportive of their therapeutic journey. Similarly, if friends or family of a low seeker consider that trying something unproven is better than not trying it, especially if they know of others or have themselves taken an unproven SCI, they may not encourage taking an unproven SCI and instead support their decision.

Few high seekers reported trusting the advice of celebrities and social media influencers aligning with previous work (37, 38). While there is rampant advertising by unproven SCI clinics providing endorsement of celebrities, athletes, and influencers (40), no studies have directly evaluated the attitudes among patients considering unproven SCIs to determine the impact celebrity endorsements have toward decision-making among low and high seekers.

## Limitations

While our study provides valuable insights into trust dynamics among US patients considering unproven SCIs, several limitations should be acknowledged. Our sample, while diverse in medical conditions and geographic distribution, may not fully represent the broader population of patients interested in experimental treatments, including women, Asian participants, and participants living in remote locations. Additional limitations of sampling have been discussed elsewhere (13). Additionally, the cross-sectional nature of interviews captures attitudes at a single point in time rather than tracking how trust relationships evolve over time. Future research should explore the temporal dynamics of (mis)trust, particularly how experiences with conventional healthcare and unproven SCI clinics shape patient trust in sources, the information they provide, and attitudes over time. As with all qualitative research, our analysis and interpretation may have been shaped by the investigative team’s prior assumptions (confirmatory bias). Although we included a reflexivity statement and took steps to minimize this influence during interviews and analysis (see Supplementary material), residual bias may still be present.

## Conclusion

Our analysis reveals that trust in the context of unproven SCIs operates as a complex, multidimensional phenomenon with distinct (mis)trust profiles among high and low seekers. Our findings suggest that trust is an important part of how patients evaluate unproven SCIs. Clinicians’ efforts to communicate with patients about these interventions may benefit from attending to existing trust relationships and social dynamics. By working with rather than against existing trust relationships and social dynamics, researchers can develop more effective approaches to protecting patients while respecting their autonomy and dignity in the face of serious illness.

## Data Availability

The raw data supporting the conclusions of this article will be made available by the authors, without undue reservation.
