# Supplementary material for: (Mis)trust among patients seeking unproven stem cell therapies: a qualitative analysis
Source: Front Med (Lausanne). 2026 May 13;13:1772881. doi: 10.3389/fmed.2026.1772881 (PMC13212192; doi:10.3389/fmed.2026.1772881)
Supplement: Supplementary file 1 [file Supplementary_file_1.docx]

**Supplemental Information**

Methodological details and justification in this Supplement have been previously reported in Kawam *et al.*  2025 or within Appendix A: Supplementary Data as part of the publication [1]. Details directly from Appendix A: Supplementary Data as part of the Kawam *et al.* 2025 article are written nearly verbatim as represented here and verbatim passages are quoted.

**Methods**

This supplemental information provides details of methods, including recruitment, the theoretical premise that forms the basis of designing the interview guide, a reflexivity statement, and the interview guide used in this research study.

**Recruitment**

The Mayo Clinic Regenerative Medicine Consult Service (RCMS) database enabled the identification of a diverse patient population for this research study. RCMS is designed to support patients by facilitating access to appropriate research studies or clinical care pathways, while also providing educational resources regarding unproven stem cell interventions (SCIs). Previous evaluations of RCMS have shown that a substantial proportion of individuals engaging with the service were new patients to Mayo Clinic, averaged 65 years of age, and were geographically dispersed across the United States of America [2, 3]. The RCMS database integrates the patient registry with electronic health records and contains sociodemographic and health information. Participant rurality was determined using zip code classifications based on the Rural-Urban Commuting Area (RUCA) codes established by the U.S. Department of Agriculture [4-6].

To categorize participants as a high or low seeker of unproven SCIs, research coordinators administered a brief screening survey consisting of three items: (i) have you heard about a stem cell procedure for your condition? (yes/no); (ii) if yes (i), have you ever taken a stem cell procedure? (yes/no).; and (iii) if (ii) no, what is the likelihood of you considering a stem cell procedure for your condition? (1-very unlikely to 5-very likely). Participants were classified as a low seeker if the individual had not received a SCI and indicated a low likelihood (ratings of 1 or 2) or pursuing one, regardless of prior awareness. High seekers were defined as individuals who had either previously obtained a SCI or expressed strong future intent (ratings of 4 or 5). Those who selected a neutral response (rating of 3) were included in the study and further probed during the interview to determine their orientation toward unproven SCIs, allowing for post hoc classification as either a low or high seeker.

**Interview Guide Development & Conducting Interviews**

To inform the development of the interview guide, we drew upon the Unified Theory of Health Behavior which aims to predict variability in deliberate behaviors based on 8 constructs– intention, environmental constraints, skills, attitudes, norms, self-standards, emotion, and self-efficacy [7]. According to the theory, intention, skills, and environmental constraints are foundational for behavior enactment while the other constructs modulate the strength and direction of intention. In the context of pursuing unproven SCIs, a patient must possess a firm commitment (intention) to undergo the intervention, the practical ability to do so (e.g., travel logistics), and the means to overcome potential obstacles (e.g. financial limitations). However, the formation of such intention is likely contingent upon a broader constellation of influences, such as favorable attitude toward a SCI (e.g., perceived risk-benefit analysis), confidence in one’s ability to follow through (self-efficacy), alignment with personal identity, perceived social endorsement of obtaining a SCI, and positive emotional valence toward a SCI. Importantly, the theory allows for asymmetry among these factors. For example, a single dominant motivator such as the belief that a SCI will offer relief from desperation may override social discouragement or a negative attitude (e.g., physician does not recommend a SCI or knowing they are at risk for severe side effects).

Interview guide questions contained questions based on the domains as well as other variables as follows: condition; belief, intention, trust, information-seeking habits; demographics. To begin, participants were invited to describe their lived experiences with the patient’s condition. Subsequent questions addressing belief, knowledge, and attitudes were informed by a comprehensive review of the empirical literature on the marketing of unproven SCIs including analyses of websites, blogs, media coverage [8-22], prospective studies on patient perceptions [2, 23-35], and investigations of social media discourse [36-38]. Additional questions were developed and tailored to the SCI context based on prior research examining how patients seek health-related information online [39-43].

The pilot interviews (two low and two high seekers) were conducted by OK, CML, and ZM. The remaining participant interviews were primarily conducted by OK.

***Reflexivity Statement – previously reported in Appendix A: Supplementary Data of Kawam et al. 2025 [1]***

“We are cognizant of our starting assumptions and biases. We started this project concerned with the extent of patient interest in unproven SCIs that have not yet been approved for use among most conditions by the U.S. Food and Drug Administration (FDA). In our view, many stem cell clinics and providers have been profiting from patients’ lack of sufficient knowledge about stem cell science and regulation and by using deceptive or misleading language through advertisements among other means. While we fully respect patient autonomy in instances where patients are suffering unbearably and where they have exhausted conventional treatment options, we believe that patients may benefit from patient-centered educational resources to properly equip them with accurate information and to support informed decision making. Working for an institution that operates on scientific and empirical evidence, we were also mindful that patients may not be aware or share the same framework for what counts as sufficient evidence to trust a procedure. As our research team represents this perspective, we aimed to separate our institutional perspective from the interview, analysis, and interpretation of results, striving instead to balance the frameworks patients have with our own, but recognizing the challenges and limits of performing such analysis in social science research.

ZM is a mid-career bioethicist and has worked on issues regarding scientific integrity, research ethics, and unproven and unregulated interventions, specifically in regenerative medicine. The interviewers (OK and ZM) did not share their values or views during the interview recognizing themselves as knowledge experts. If participants brought up specific “conspiracy” or “anti-establishment” viewpoints, the interviewers permitted the flow of information without sharing their own views. Many patients considered interviews as an opportunity to share their personal health journeys, frustrations accessing medical treatments, trust in specific individuals and organizations,nand feelings of hopelessness and desperation in pursuit of care for themselves or loved ones. Interviewers strove to respect patient experiences providing emotional comfort as needed while not compromising the integrity of the interviews. Both interviewers created a comfortable atmosphere by encouraging participants to voice opinions and did not judge responses. OK was a post-baccalaureate research trainee interested in ethical issues related to health information and patient decision making and served as primary coder. XZ served as the secondary coder and has expertise in qualitative research and patient health behaviors. As colleagues, both coders were committed to challenging one another and trying to work against any ethical biases that may have impacted data collection, analysis, and reporting.”

***Interview Guide – previously reported in Appendix A: Supplementary Data of Kawam et al. 2025 [1]***

**“Preamble*:*** (read to participants)

I’m going to be asking you several questions about your health condition and about stem cell procedures. There are many clinics in the US and worldwide that are giving stem cell procedures and some might question them. We want to know what you think of these and other stem cell procedures.

Interested in asking questions about your experiences with stem cells and in particular ones given outside of a hospital setting.

Please understand that there are no right or wrong answers to any of these questions. I am interested in your opinions and views.

**(A) Condition**

1) Please tell me a bit about your condition, how long have you had it, some of the challenges with living with it.

2) Are you looking/ have you looked for an experimental procedure/therapy for your condition? What kinds of procedures/therapies have you looked into?

**(B) Beliefs Toward Stem Cell Procedures**

3) What do you know about stem cell procedures for your condition?

4) Where did/does stem cells fit in your search for treatment for your condition? (What priority do you give a stem cell procedure over another experimental treatment?)

5) What are the kinds of stem cell procedures you’ve investigated (hospital/medical center vs for-profit clinic)?

Triage participant into 3 groups:

1. Only interested in legitimate clinical trials
2. Interested in for-profit stem cell interventions but have NOT taken one
3. Interested in for-profit stem cell interventions and have taken one

**Note:** Because participants may conflate legitimate research/clinical trials with for-profit stem cell interventions, we will assess their responses and modify questions according to the above categories and their knowledge of clinical trials and the for-profit stem cell industry.

- 1. If they answer Q5 with just clinical trials, ask if they know about for-profit industry.
     1. If they do not know about for-profit stem cell industry, make a note, and only ask questions about their searching behaviors, trust in scientific/medical establishment, and trust in regulations (skipping the rest of this section-Section B).
     2. If they do know about the for-profit industry and have avoided it, make a note and ask questions about why they have avoided it, and then ask questions about their searching behaviors, trust in scientific/medical establishment, and trust in regulations.
  2. In the likely chance that participants mix between for-profit and clinical trials, we can ask them what they think is the difference is (b,i) so we know what their terminology means, and proceed with the interview guide below.
     1. What do you think are the similarities and differences between <Clinical Trial at University/Hospital> and <For-Profit Clinic>?
        1. If they conflate the two (e.g., that they “both use stem cells” and are the same) or don’t know the important differences (i.e., trials have regulatory oversight and clinics don’t), we should take note of their terminology/understanding and proceed with interview guide as below, no changes needed.
        2. At the end of the interview, we will explain the difference and ask a set of questions given their new knowledge (see section (G) “Afterwards”).
  3. If they already underwent a stem cell intervention at a for-profit clinic, we will:
     1. ask them similar questions as in (b).
     2. slightly modify interview guide to ask them to retrospectively reflect on their experiences before, during, and after getting the intervention.
        1. E.g.,: What did you think were the advantages of getting an unproven SC treatment at the for-profit clinic? What did you think the advantages were during the procedure? Now?

***Behavioral Beliefs – Perceived consequences of undertaking/not unproven stem cell procedures***

6) Do you think that there are any advantages of taking an unproven/ for profit stem cell procedure for your condition? If so, what are they?

*Possible Probes:*

- Curing illness; relief of symptoms; reducing medication
- Finding “hope” (not giving up)
- Gaining control over illness

7) Do you think that there might be any disadvantages of taking a stem cell procedure for your condition? If so, what are they?

*Possible Probes:*

- Nothing happens; get worse symptoms or new symptoms; need more medication or other medication
- Losing “hope” (giving up)
- Losing money
- Losing control to illness

8) What specifically would you want to have happen to you if you took a stem cell procedure for your condition?

*Possible Probes:*

- Curing illness; relief of symptoms; reducing medication
- Finding “hope” (not giving up)
- Gaining control over illness

9) What specifically would you not want to have happen to you if you took a stem cell procedure for your condition?

*Possible Probes:*

- Nothing happens; get worse symptoms or new symptoms; need more medication or other medication
- Losing “hope” (giving up)
- Losing money
- Losing control to illness

***Normative Beliefs – Approval/disapproval of taking/not an unproven stem cell procedure by others***

10) Who believes it is a good idea for you to have a stem cell procedure for your condition? *(Identify Specific People)*

*Possible Probes:*

- Family members; friends; clinicians; pastor/priest
- Unproven/ for-profit SCI provider(s)
- Patient group
- Specific communities
- Treatment physicians

11) Will you go and get a stem cell procedure if your family/friends/patient group/doctor says it is a good idea? Why?

*Possible Probes:*

- Identify whose opinion matters: family members; friends; clinicians; pastor/priest, unproven/ for-profit SCI provider, patient group, treatment team

12) Who believes it is a bad idea for you to have a stem cell procedure for your condition?

*Possible Probes:*

- Family members; friends; clinicians; pastor/priest
- Unproven for-profit SCI provider(s)
- Patient group
- Specific communities
- Treatment physicians

13) Will you go and get an unproven for-profit stem cell procedure, if you wanted to but your family/friends/patient group/doctor says it is a bad idea? Why?

*Possible Probes:*

- Identify whose opinion matters: family members; friends; clinicians; pastor/priest, unproven SCI provider, patient group, treatment team

***Control Beliefs – factors that facilitate/inhibit the likelihood of undergoing an unproven*** for-profit ***SCI***

14) Are there any circumstances that would make it difficult for you to go and get a stem cell procedure for your condition?

*Possible Probes:*

- My symptoms worsen
- Cost: I had other financial obligations or the SCI became more expensive
- If my doctor said it was harmful to try it (or other specific people)

15) Are there any circumstances that would make it easier for you to go and get a stem cell procedure for your condition?

*Possible Probes:*

- My symptoms got better
- Cost: I ran into more money or the SCI was cheaper than I thought
- If my doctor said it was good to try it (or other specific people)

16) Is cost an issue for you in getting a stem cell procedure?

17) What info would make you change your mind?

Probes would be to list things like examples of

- serious harms
- lawsuits
- Payments
- no efficiency,
- Outside any oversight (although we do somewhat cover this)

**(C) Intentions**

18) How likely is it that you would undergo a stem cell procedure for your condition if you could today?

**(D) Knowledge**

19) Is it important for you to know whether a stem cell procedure is approved by the U.S. Food and Drug Administration (FDA)? Why/why not?

*Possible Probe:*

- You may inform participant what the FDA does (note it)

*(For those who have contacted a clinic, done research, or have undertaken an unproven (for profit) stem cell procedure)*

20) What do you know about the stem cell procedure you received/found out about?

*Possible Probes:*

- Is this information important for you to know? Why/why not?
- Will this information affect your desire to go and get an unproven (for profit) stem cell procedure? In what ways?

21) Do you think the stem cell procedure you will get is safe? Why/why not?

22) Do you think the stem cell procedure you will get is effective? Why/why not?

*Possible Probes:*

- Is this information important for you to know?
- Will this information affect your desire to go and get an unproven (for profit) stem cell procedure?

23) Do you think stem cells taken from your body are safer than ones you get from others? (Why?)

24) Have you heard about any warnings about stem cell procedures?

*Possible Probes:*

- Where
- From whom (doctor, friend, family, patient advocacy group)
- What sources (online text, video)

25) Is it important for you to know what qualifications the doctors who give stem cell procedures have?

*Possible Probes:*

- Yes – what qualifications are important to you? How do you find out if the doctor has this qualification?
- No/Not really – why?

26) Do you think it is important for scientists to show if a stem cell procedure is safe and effective from patient trials (studies) before you get it? (Why or why not?)

*Possible Probes:*

- If so, what info is pertinent, stem cell biology, the regulatory process, how long it takes to get a drug to market?
- Does the participant think the same stem cell can treat multiple conditions?
- Does the participant think the same procedure to get stem cells into their body will treat every kind of disease?

27) A stem cell procedure that is experimental means it is not fully known if it will be safe or effective at treating your disease. Do you think clinics giving a stem cell procedure are experimental?

*Possible Probes:*

- Find out if participant is familiar with regulatory process, including trial registration, need for ethics or FDA approval

28) Ethics boards examine stem cell procedures to make sure you are properly informed about potential risks, benefits, and what you should expect from a stem cell procedure. How important is it for you to know whether a stem cell procedure was approved by an ethics board?

**(E) Trust in Scientific and Medical Establishments**

29) How much would you trust the information scientists provide on stem cell procedures? Why?

30) Do you believe that scientists are doing all they can to help make discoveries to treat your condition?

31) How much would you trust the information from your current doctors? Why?

32) How much would you trust the information provided to you by doctors working at a stem cell clinic? Why?

33) Do you believe the Food and Drug Administration helps to make sure stem cell procedures are safe and effective?

34) Do you think politics plays a role in getting stem cell treatments to you?

35) What are the trusted bodies you refer to for guidance?

**(F) Information Seeking Habits: *Online, Interpersonal, Other***

36) How have you found out about stem cell procedures for your condition?

*Possible Probes:*

- Probe to find out if they first heard from someone and then searched. Who was most influential?
- Find out if they looked up scientific articles, news articles or other sources
- Was there a google/yahoo/bing search done? Keywords?

(*For those that go online to do research)*

37) When you go online, do you just search for things or do you go to specific websites?

*Possible Probes:*

- This could include websites, blogs (expert/patient), discussion forum, reading posts/comments, private messaging.

38) Is there a particular website for health information you go to? (Read each one and check off which they recall)

| WebMD | CDC | Mayo Clinic | KidsHealth | NIH | Yahoo! Health |
| --- | --- | --- | --- | --- | --- |
| Drugs.com | WeightWatchers | Med Society of _________ | Healthline | Everyday Health | MedicineNet |
| Medscape |  |  |  |  |  |

39) Which specific warnings have you looked at? (Read each one and check off: ISSCR, FDA, CDC, WebMD, Medscape, Healthline, Mayo Clinic, NIH)

40) What impact did the warnings have on you if at all?

41) What kind of warning would make you rethink taking an unproven SC from a for profit clinic?

42) What forms of social media do you use and do you use it to connect or find out about stem cell procedures?

| YouTube | Facebook | Instagram | Twitter | Pinterest | GoFundMe |
| --- | --- | --- | --- | --- | --- |
| PatientsLikeMe | LinkedIn | Snapchat | WhatsApp | Reddit |  |

*Possible Probes:*

- If participants reports spending more time on one site, ask about how they search, why they think it is a good website/SM platform, and what info they look for

43) How much time do you think you spend searching for online information?

44) You mentioned ______, ______, ______ sources that you particularly visit. For each of these, can you tell me what you think about the information you found?

*Possible Probes:*

- Probe to find out why they find the source credible: believable, trustworthy, accurate, truthful, complete/thorough, unbiased.

45) Are there specific people that have helped you find information or alerted you to some online information?

*Possible Probes:*

- Could be spouse/partner, family member, caregiver, doctor, another patient

**(G) Demographics (Already Collected):**

**(H) Attitudes of For-Profit Clinics After Explanation**

*For patients who conflated clinical trials and for-profit clinics, we will need to ask them a few final questions after we explain the difference between the two.*

Clinical Trials are often free, undergo ethical review, and have oversight by FDA and abide by regulations. They aim to collect information, monitor patients, and report data in scientific publications.

For-profit clinics often are not experimental because unlike clinical trials, they are not doing research to better understand how safe and effective stem cell treatments are. They may advertise their stem cell procedures as a clinical trial, or as a therapy or treatment. They often don’t have a good scientific reason to back their claims. The people giving the treatments are not trained in the specific procedure or field of medicine being treated. Clinics rely on using patient testimonials and advertise the effectiveness of the treatments. They usually accompany their treatments with many additional therapies and are often expensive. They are also not regulated by the FDA because they are not clinical trials. There have also been many harms that have occurred when patients receive for-profit stem cells, including infection, tumor growth, and other complications. If something happens, providers at for-profit clinics do not try and assess or take care of you.

46) Given this explanation:

1. How, if at all, does this change your view about getting a for-profit stem cell treatment?
2. Would you change your intentions to seek a for-profit stem cell treatment?
3. What would change your intention to seek a for-profit stem cell treatment?

*Thank you so much for taking the time to talk with us. We really appreciate it.”*

**Qualitative Analysis**

Qualitative analysis was done by OK and reviewed by XZ and ZM. Data were analyzed using a constant comparative method informed by grounded theory using NVivo 14 software (QSR International). Initial descriptive coding was followed by iterative cross-case comparisons to identify emergent patterns in beliefs, attitudes, intentions, emotional responses, and sources of online information. For interviews involving family members, a tailored analytic strategy was employed: each individual’s responses were coded independently, then examined both within familial units and across families [44]. This multi-layered approach enabled a nuanced exploration of intra- and inter-family dynamics, including tensions and asymmetries in perspectives between patients and their relatives.

**References**

1. Kawam, O., et al., *Factors that influence unproven stem cell intervention seeking behavior: A qualitative analysis of U.S. patients considering or having undertaken unproven stem cell interventions.* Social Science & Medicine, 2025: p. 117795.

2. Smith, C., et al., *Challenging misinformation and engaging patients: characterizing a regenerative medicine consult service.* Regenerative Medicine, 2020. **15**(3): p. 1427-1440.

3. Arthurs, J.R., et al., *Patients seeking stem cell therapies-a prospective qualitative analysis from a Regenerative Medicine Consult Service.* NPJ Regenerative Medicine, 2022. **7**(1): p. 20.

4. Morrill, R., J. Cromartie, and G. Hart, *Metropolitan, urban, and rural commuting areas: toward a better depiction of the United States settlement system.* Urban Geography, 1999. **20**(8): p. 727-748.

5. Hart, L.G., E.H. Larson, and D.M. Lishner, *Rural definitions for health policy and research.* American Journal of Public Health, 2005. **95**(7): p. 1149-1155.

6. University of Washington. *Rural Urban Community Area Codes Maps*. Available from: depts.washington.edu/uwruca/ruca-maps.php.

7. Fishbein, M., et al., *Factors Influencing Behavior and Behavior Change*, in *Handbook of Health Psychology*, B. AS, T.A. Revenson, and S. JE, Editors. 2001, Lawrence Erlbaum: Mahwah, New Jersey. p. 3-17.

8. Bauer, G., M. Elsallab, and M. Abou‐El‐Enein, *Concise Review: A Comprehensive Analysis of Reported Adverse Events in Patients Receiving Unproven Stem Cell-Based Interventions.* Stem Cells Translational Medicine, 2018. **7**(9): p. 676-685.

9. Berger, I., et al., *Global Distribution of Businesses Marketing Stem Cell-Based Interventions.* Cell Stem Cell, 2016. **19**(2): p. 158-162.

10. Chang, W., T.C. Bank, and C.T. Scott, *Fit to print? Media accounts of unproven medical treatments across time.* AJOB Empirical Bioethics, 2014. **5**(1): p. 33-43.

11. Kingery, M.T., et al., *Online Direct-to-Consumer Advertising of Stem Cell Therapy for Musculoskeletal Injury and Disease: Misinformation and Violation of Ethical and Legal Advertising Parameters.* The Journal of Bone and Joint Surgery, 2020. **102**(1): p. 2-9.

12. Lau, D., et al., *Stem cell clinics online: the direct-to-consumer portrayal of stem cell medicine.* Cell Stem Cell, 2008. **3**(6): p. 591-594.

13. Marcon, A.R., B. Murdoch, and T. Caulfield, *Fake news portrayals of stem cells and stem cell research.* Regenerative Medicine, 2017. **12**(7): p. 765-775.

14. Munsie, M., et al., *Open for business: a comparative study of websites selling autologous stem cells in Australia and Japan.* Regenerative Medicine, 2017. **12**(7): p. 777-790.

15. Murdoch, B., A. Zarzeczny, and T. Caulfield, *Exploiting science? A systematic analysis of complementary and alternative medicine clinic websites' marketing of stem cell therapies.* BMJ Open, 2018. **8**(2).

16. Ogbogu, U., J. Du, and Y. Koukio, *The involvement of Canadian physicians in promoting and providing unproven and unapproved stem cell interventions.* BMC Medical Ethics, 2018. **19**(1): p. 32.

17. Petersen, A., C. MacGregor, and M. Munsie, *Stem cell miracles or Russian roulette?: patients’ use of digital media to campaign for access to clinically unproven treatments.* Health, Risk & Society, 2016. **17**(7-8): p. 592-604.

18. Rachul, C., *"What have I got to lose?": an analysis of stem cell therapy patients' blogs.* Health L. Rev., 2011. **20**: p. 5.

19. Regenberg, A.C., et al., *Medicine on the fringe: stem cell-based interventions in advance of evidence.* Stem Cells, 2009. **27**(9): p. 2312-2319.

20. Ryan, K.A., et al., *Tracking the rise of stem cell tourism.* Regenerative Medicine, 2010. **5**(1): p. 27-33.

21. Turner, L. and P. Knoepfler, *Selling stem cells in the USA: assessing the sirect-to-consumer industry.* Cell Stem Cell, 2016. **19**(2): p. 154-157.

22. Zarzeczny, A., et al., *Stem cell clinics in the news.* Nature Biotechnology, 2010. **28**(12): p. 1243-1246.

23. Brophy, J., *Navigating the ‘grey areas’: Australian medical travellers in China's stem cell bionetwork.* Asia Pacific Viewpoint, 2017. **58**(2): p. 216-227.

24. Chen, H. and H. Gottweis, *Stem cell treatments in China: rethinking the patient role in the global bio-economy.* Bioethics, 2013. **27**(4): p. 194-207.

25. Kenihan, L., L. McTier, and N.M. Phillips, *Patients' expectations and experiences of stem cell therapy for the treatment of knee osteoarthritis.* Health Expectations, 2020. **23**(5): p. 1300-1309.

26. Kim, Y.S., et al., *Fantasies About Stem Cell Therapy in Chronic Ischemic Stroke Patients.* Stem Cells and Development, 2013. **22**(1): p. 31.

27. Miles, J. *Sick warned against falling for ‘stem cell’ tourism*. 2008.

28. Petersen, A., K. Seear, and M. Munsie, *Therapeutic journeys: the hopeful travails of stem cell tourists.* Sociology of Health & Illness, 2014. **36**(5): p. 670-685.

29. Petersen, A., C. Tanner, and M. Munsie, *Navigating the cartographies of trust: how patients and carers establish the credibility of online treatment claims.* Sociology of Health & Illness, 2019. **41**: p. 50-64.

30. Prasad, A., *Ambivalent journeys of hope: embryonic stem cell therapy in a clinic in India.* Health (London), 2015. **19**(2): p. 137-153.

31. Song, P., *Biotech pilgrims and the transnational quest for stem cell cures.* Medical Anthropology, 2010. **29**(4): p. 384-402.

32. Tanner, C., A. Petersen, and M. Munsie, *'No one here's helping me, what do you do?': addressing patient need for support and advice about stem cell treatments.* Regenerative Medicine, 2017. **12**(7): p. 791-801.

33. Unsworth, D.J., et al., *Stroke survivor attitudes toward, and motivations for, considering experimental stem cell treatments.* Disability and Rehabilitation, 2020. **42**(8): p. 1122-1130.

34. Vicsek, L. and J. Gergely, *Media presentation and public understanding of stem cells and stem cell research in Hungary.* New Genetics and Society, 2011. **30**(1): p. 1-26.

35. Waldby, C., et al., *The direct-to-consumer market for stem cell-based interventions in Australia: exploring the experiences of patients.* Regenerative Medicine, 2020. **15**(1): p. 1238-1249.

36. Hawke, B., et al., *How to peddle hope: an analysis of YouTube patient testimonials of unproven stem cell treatments.* Stem Cell Reports, 2019. **12**(6): p. 1186-1189.

37. Kamenova, K., A. Reshef, and T. Caulfield, *Representations of stem cell clinics on Twitter.* Stem Cell Reviews and Reports, 2014. **10**(6): p. 753-760.

38. Robillard, J.M., et al., *Fueling hope: stem cells in social media.* Stem Cell Reviews and Reports, 2015. **11**(4): p. 540-546.

39. Antheunis, M.L., K. Tates, and T.E. Nieboer, *Patients' and health professionals' use of social media in health care: motives, barriers and expectations.* Patient Education and Counseling, 2013. **92**(3): p. 426-431.

40. Flanagin, A.J. and M.J. Metzger, *perceptions of internet information credibility.* Journalism & mass communication quarterly, 2000. **77**(3): p. 515-540.

41. Griffiths, F., et al., *The impact of online social networks on health and health systems: a scoping review and case studies.* Policy Internet, 2015. **7**(4): p. 473-496.

42. Mironova, E., *Audience's behavior and attitudes towards lifestyle video blogs on Youtube*, in *Faculty of Culture and Society*. 2016, Malmo University.

43. Pew Research Center. *Social Media Fact Sheet*. 2021 April 7; Available from: https://www.pewresearch.org/internet/fact-sheet/social-media/.

44. Van Parys, H., et al., *Multi family member interview studies: A focus on data analysis.* Journal of Family Therapy, 2017. **39**(3): p. 386-401.
